# Dual Implantation of Artificial Urinary Sphincter and Inflatable Penile Prostheses for Concurrent Male Urinary Incontinence and Erectile Dysfunction

**DOI:** 10.1155/2011/178312

**Published:** 2011-11-17

**Authors:** Tariq F. Al-Shaiji

**Affiliations:** Toronto Western Hospital, University Health Network, Toronto, ON, M5T 2S8, Canada

## Abstract

Erectile dysfunction and urinary incontinence secondary to sphincter dysfunction are common conditions affecting many men worldwide with a negative effect on quality of life. They are encountered in a number of etiologies most commonly following radical prostatectomy in which they coexist in the same patient. Implantations of an artificial urinary sphincter and inflatable penile prosthesis have proven to be effective in the treatment of both conditions should conservative and minimally invasive measures fail. The recent literature has shown that dual implantation of these devices is feasible and safe with a durable clinical outcome. Once indicated, this can be done in a synchronous or nonsynchronous manner; however, the emerging of the single transverse scrotal incision as well as advancement in the prostheses has made synchronous dual implantation more favourable and appealing option. It provides time and cost savings with an evidence of high patient satisfaction. Synchronous dual implantation should be offered initially when indicated. This paper discusses the surgical techniques of artificial urinary sphincter and inflatable penile prosthesis dual implantation in the management of concurrent moderate-to-severe urinary incontinence and medically refractive erectile dysfunction, in addition to highlighting the existing literature pertaining to this approach.

## 1. Introduction

Erectile dysfunction (ED) is defined as “the consistent inability to achieve and maintain a penile erection adequate for satisfactory sexual intercourse” [1]. It has been estimated that 150 million men worldwide had some degree of ED in 1995 and that approximately 322 million men are expected to be affected by 2025 [2]. Once considered primarily a psychogenic disorder, ED now is recognised to have a well-established association with a variety of organic causes with radical prostatectomy being one of the leading causes. It has been suggested that the mean potency rate after radical prostatectomy in general urologic practice is only 19%, regardless of surgical technique used [3]. There are subgroups of ED patients who do not respond well to first- and second-line therapies. In these men inflatable penile prosthesis (IPP) implantation provides an excellent surgical option with a high satisfaction rate.

Stress urinary incontinence (SUI) refers to the involuntary leakage of urine which occurs during activities that exert pressure on the bladder. SUI in men most commonly results from prostate surgery leading to intrinsic sphincteric deficiency. SUI due to urethral sphincter dysfunction affects up to 40% of men who have undergone radical prostatectomy [4]. The artificial urinary sphincter (AUS) has found widespread use in males with SUI and is currently considered the gold standard management option for men with postprostatectomy incontinence with a success rate of over 90% [5].

There are a significant number of men in which ED and SUI coexist, especially in the postprostatectomy population that contributes to a significant decline in quality of life. In a study by Wille et al., data on 327 men who had undergone radical prostatectomy was presented. The authors found a statistically significant higher rate of incontinence among men who had poor erectile function both before and after surgery [6]. Some men who present with urinary incontinence tend to be so much bothered by this problem that they overlook their concomitant ED, and, therefore, men considering sphincter implantation should undergo careful questioning about their sexual function, and vice versa, before any intervention.

Should conservative treatment modalities fail in these patients, synchronous or nonsynchronous insertion of an AUS and IPP is an appropriate treatment option, with good long-term results. Historically, AUS implantation has required two incisions and IPP was included during staged and/or simultaneous implantation [7]. Simultaneous implantation was met with some skepticism in the past. Nevertheless, since Wilson et al. introduced transscrotal placement of an AUS in 2003 [8], dual implantation of an AUS and IPP has been shown to constitute a safe and efficient method to treat both morbidities through a single incision. Patients and surgeons should realize that to combine a second implant during the initial implant surgery is neither complicated nor hazardous.

## 2. Dual Prosthetic Implantation

Dual implantation of AUS and IPP can be accomplished either simultaneously or as a two-stage procedure. This has been made possible by significant improvements in the surgical implantation techniques as well as the mechanical properties and functional capabilities of these devices. As far as dual implantation is concerned, recent trends have favoured synchronous over nonsynchronous insertion [9–12]. Synchronous dual implantation may be indicated as a primary procedure in patients who are refractory to conservative management of ED and SUI or may be required during revision for one device and de novo placement of the second device. Comparison between advantage and disadvantage of synchronous and nonsynchronous dual prosthetic insertion is shown in Table 1.

## 3. Surgical Technique

### 3.1. Synchronous Dual Implantation

The patient is placed supine under general anaesthesia with the legs slightly abducted (modified low lithotomy position) when a single incision procedure is planned. This position allows complete access to the bulbar urethra and the corpora. In case a twin incision approach is planned where an incision in the perineum is to be used to place the AUS cuff, a classic lithotomy position will be required to access the perineum. He should be shaved in the operating room, just prior to surgery. The patient is scrubbed for 10 minutes with povidone-iodine or chlorhexidine scrub, followed by application of alcohol-based disinfectant before draping in sterile fashion. Recently, Darouiche et al. showed that preoperative cleansing of patient's skin with chlorhexidine-alcohol is superior to cleansing with povidone-iodine for preventing surgical-site infection after clean-contaminated surgery [18]. Next, the bladder is completely drained via a 12–14 Fr Foley catheter which should remain indwelling to facilitate dissection and identification of the urethra. It is universally accepted that broad-spectrum antibiotics covering both aerobic and anaerobic organisms should be administered perioperatively. 

A double circle Scott retractor is placed around the genitals with the penis pointing cephalad in the larger ring. A modification of the Scott retractor was introduced by Wilson et al. to facilitate the scrotal incision [19], the so-called SKW Scrotal Retraction System. It consists of a sharp hook for placing the penis on stretch, seven blunt hooks to secure the scrotal incision upon the penoscrotal area, a self-retaining retractor, a penile strap, two plastic baby Deavers, and two large rake hooks. A 3-4 cm transverse scrotal incision is fashioned a few centimetres below the penoscrotal junction and deepened through the subcutaneous tissue. Blunt stay hooks are placed at the 1, 3, 5, 7, 9, and 11 o'clock positions to secure the scrotal incision and expose the corpus spongiosum and the 2 corpora cavernosa. The stays are repositioned as deeper layers of tissue are dissected. The dissection of the corpus spongiosum of the bulbous urethra involves careful division of the scrotal septum. This incision may limit access to the bulbar urethra and may require significant retraction for accurate exposure. An alternative approach involves two incisions [7]. A perineal incision is made to place the AUS cuff, and a second infrapubic incision is used to place the rest of the AUS components and all components of the IPP.

Insertion sequence of either the sphincter cuff or the prosthesis cylinder first is surgeon dependent. Nevertheless, since cuff placement requires circumferential mobilization of the urethra at the bulb which includes dissection at the level of the corporal bodies, it is recommended that mobilization be performed first because injury to the urethra would require abandoning or modifying the procedure without discarding any of the prosthesis [13]. Once the cuff is in place, corporotomies can follow. On the other hand, patients requiring replacement of a penile prosthesis and placement of a de novo AUS should have the IPP cylinders replaced before inserting the AUS cuff [13].

Dividing the scrotal septum exposes the corpus spongiosum which is enclosed by the bulbocavernosus and bulbospongiosum muscles. To expose the bulbar urethra, these muscles must be carefully dissected. The use of two infant Deavers will maximize exposure and facilitate dissection. Alternatively, the SKW Scrotal Retractor System can be applied in which placing the rakes deep in the scrotal tissue at 4:00 and 8:00 duplicates the exposure obtained by the infant Deavers and thus eliminates the awkwardness of an assistant holding the Deavers constantly. Wilson et al. suggested that placing a rolled Raytec sponge “the cowboy blanket roll” under the rakes acts to widen their retraction and further improves deep bulbar urethra access that can be made even more better by placing a weighted “swan neck” vaginal speculum over the rakes [19]. Curved Metzenbaum scissors are passed just lateral to the ventral side of the spongiosum which are used to open Bucks fascia at both sides of the bulbar urethra that is separated from the corpora cavernosa. A right angle clamp is used for further careful blunt dissection of the spongiosum from Buck's fascia. Circumferential dissection of the urethra is performed for a sufficient vertical distance to accommodate the 2.0 cuff width. During mobilization of the bulb, the dissection should be performed outside of the corpus spongiosum which should be included within the cuff to minimize chances of urethral injury and erosion. A safety check many surgeons apply at this point is to remove the Foley catheter and shot fluid down the urethra in a retrograde fashion; if fluid extravates at the dissection site, there is a urethral injury. The bulbar urethral circumference is measured using the cuff sizer. A right angle clamp is used at the end of this careful dissection to facilitate the posterior circumferential dissection and grabs the cuff sizer tape. The AUS is then placed at the ventral surface of the bulbar urethra. It is essential to avoid denuding the spongiosus muscle bulb during the cuff placement. An absorbable suture is used to close the dartos over the cuff. The surgeon must bear in mind that two pumps and two sets of tubing will exit from the scrotum and that the cuff tubing must not cross. Wilson et al. reported that the bulbocavernosus muscle does not need to be disturbed when placing the AUS cuff around the bulb when using the single upper transverse scrotal incision [8]. However, in order to access the proximal bulb, the muscle must be retracted ventrally while continuing to divide the midline attachment to the raphe. This dissection should be continued until the perineal body has been encountered and divided sharply. 

The next step is to fashion vertical corporotomies bilaterally using the same scrotal incision. These are typically made 1 cm lateral to the corpus spongiosum. Corporal dilatation then proceeds cautiously proximally and distally followed by length measurement using a measuring device such as the Dilamezinsert device. Attention must be drawn to avoid perforation of the distal or crural ends of each corpus cavernosum, crossing over, injury to the urethra or the cuff. At the level of the bulbar urethra, the corporal bodies have already diverged, and therefore the likelihood of injuring the cuff or urethra is lower. The erectile tissue is lavaged with antibiotic solution then properly sized cylinders are inserted bilaterally. The corporotomies are closed such that they are water tight. Preplacement of the corporotomies sutures prior to the cylinders may be performed to decrease the risk of puncture. Next the bladder is emptied by suction in preparation for the placement of the AUS pressure regulating balloon (PRB) and the IPP reservoir in the prevesical Retzius space, one on each side. To do this the floor of the inguinal canal (transversalis fascia) is pierced (immediately above the pubic bone) either bluntly or with scissors, and the space is entered bilaterally. Infant Deaver is used to pull the external inguinal ring cephalad. Further finger blunt dissection behind the pubic symphysis is often needed to prepare the space for receiving either the PRB or the reservoir. The use of nasal speculum can facilitate the placement of the PRB and the reservoir within the retropubic space. Once they are in place, the hydraulic systems are filled with sterile saline, and the tubing, pump, and connections are completed. The inferior aspect of the scrotal incision is elevated, and two subdartos pouches are created, one on each side. Each control pump is placed within its ipsilateral space so that it sits in an easily palpable, dependent scrotal position for activation and deactivation. During the placement, the tubing must exit straight toward their pump without entangling or crossing over. A purse-string suture around the opening of the tunnel is loosely tied to secure each pump position. It is strongly advised to separate the two devices with a “wall” of dartos between the implants to try to compartmentalize each device separately. Absorbable suture is used to close the dartos and scrotal skin, with care not to damage any prosthetic material.

A suction drain is not commonly used; however, if deemed needed, then it may be placed at the lowest point of scrotal dissection, coming out at the level of the pubic tubercle. The IPP is tested and left inflated overnight to minimize bleeding. A “mummy wrap” can be used to decrease hematoma formation [20]. The AUS is cycled and left in a deactivated state. Meticulous care and avoidance of hematoma formation is an important maneuver to help avoid infection postoperatively. Some authorities apply ice packs to the perineal area to reduce edema and assist with pain control [7]. A Foley catheter is left in place for 24 hours, and the patient is observed in the hospital overnight in which another dose of intravenous broad-spectrum antibiotics should be given. The patient is discharged home on oral antibiotics and analgesia. The AUS is activated after 6 weeks when the IPP cycling can also begin.

### 3.2. Sequential Dual Implantation

If the implants are to be placed nonsynchronously, the AUS is usually placed first followed by the IPP. Most authors report that it is easier to do the AUS portion of the insertion first, then start placing the penile components [7, 9]. Great care is needed to avoid interrupting the preexisting device and its tubing. It is universally accepted that all implants will have ipsilateral placement of the tubing, pump, and PRB/reservoir which can be confirmed by reviewing the surgeon's operative notes. Nevertheless, to avoid any surprises or when in doubt, a preoperative radiograph or magnetic resonance imaging study can be utilized to confirm the location. Once the side is ratified, the second device's pump and PRB/reservoir can be planned for placement on the contralateral side. To make an incision that is located away from the existing device and to use a cautery at cutting currents during dissection will help to avoid damaging to the preexisting device and its tubings [7].

## 4. Clinical Outcomes

Generally there is a relative paucity of data involving the outcome of dual insertion of AUS and IPP, and only few centers have been performing the procedure. It has often been considered hazardous and was not routinely recommended. Nevertheless, improvements in surgical technique, surgeon expertise, and devices construction have made this approach more popular. 

From a nonpublished personal data, two patients consented for and underwent synchronous dual insertion (AMS 800 AUS and AMS 700CX IPP) via a single trans-scrotal technique. In both cases, the AUS cuff was placed first followed by implantation of the IPP. Following a mean followup of 10 months, no infection or erosions were encountered. In addition, both men considered the outcome as satisfactory, although one patient expressed some difficulties operating both systems at the beginning which resolved with education and practice. 

One of the first published series of dual implantations was reported by Parulkar and Barrett [7]. Of 65 patients who had concurrent implantation of the AUS (either the earlier AMS 721 or the newer AMS 800) and various categories of penile prosthesis (ranging from semirigid rods to three-piece IPP), 60 were followed over a mean of 35 months. In 14 patients, the penile prosthesis implant followed the sphincter implant, in 11 patients it preceded the sphincter implant, and in 40 cases the two were simultaneously implanted. Continence was graded as good or satisfactory in 95% of the patients and poor in 5%. The penile implants were functional in 98% of the patients. Of the 60 patients 33 required 59 corrections, for an average of 0.98 corrections per patient. Seven patients required devices removal because of infection: 2 AUS alone, 2 penile prosthesis alone, and 3 AUS and penile prosthesis. Further 3 patients had both sets of components removed for erosion of the cuff or cylinders. The overall erosion/infection rate was 11%. The AUS mechanical and technical failure rate was 48% with a proportionally higher rate of failure in the group where the older AUS device was used (62% versus 25%). None of the patients who received the currently available AMS 700CX penile prosthesis required a revision as the device worked well. In their series, the authors utilized a twin incision approach for their simultaneous implants with the AUS placement taking place first prior to the corporotomy for inserting the penile prosthesis. 

Marks and Light reported a total of 37 patients who underwent implantation AUS for urinary incontinence after prostatectomy in which 4 of these men had a synchronous insertion of an AUS and penile prosthesis [21]. After a mean of 37-month followup, social continence was achieved in 94.5% of the patients. None of the 4 patients who underwent the dual implantation had any complication. 

Wilson and Delk elegantly described a novel single upper transverse scrotal incision to insert an AUS [22]. In a subsequent publication, Wilson et al. reviewed their results of this technique [8]. A total of 37 patients have had AUS (AMS Sphincter 800) insertion using the new technique for revisions or reimplantations of a sphincter previously removed for infection/erosion (12) or as an initial procedure (25). In 9 of the 25 patients and 2 of the 12 dual implantations of a 3-piece penile prosthesis through the same incision was carried out. At a mean followup of 12 months, 1 patient developed early penile prosthesis infection requiring its removal. The AUS was not infected and did not require simultaneous removal. The penile implant was replaced 6 months later without disturbing the sphincter. They also described 1 case of a previous penile implant in which iatrogenic laceration of the urethra occurred during mobilization, and the procedure was aborted. Overall, the incidence of infection was 9%, and no patient had mechanical failure or atrophy at one year.

Kendirci et al. performed a multi-institutional, retrospective analysis in patients undergoing dual AUS and IPP implantation (AMS 800 AUS and AMS 700CX IPP) via a single transscrotal technique [9]. A total of 22 men underwent dual implantation between 2000 and 2003 in a synchronous manner. The implant procedure began with placement of the AUS cuff around the bulbar urethra followed by implantation of the IPP. Over a mean followup of 17 months, there were urethral erosion in 2 patients (9%) and reservoir migration in 2 (9%). None of the patients experienced any prosthetic infection postoperatively. The overall revision rate was 14% which was related to urethral erosion of the AUS device in 2 patients and to reservoir migration in 1 patient. Urine leakage decreased from a mean of 6 to 1 or fewer pads per day. 

Sellers et al. were the first to evaluate the efficiency, safety, and cost effectiveness of synchronous prosthetic implantation using a single transverse scrotal incision [11]. They compared the operative times and outcomes among 3 groups in 1 center during a 28-month period: 92 IPP patients, 21 AUS patients, and 15 dual IPP/AUS patients. Of the 128 patients, 105 received rifampin/minocycline-impregnated penile prostheses (AMS 700CX Inhibizone) and 2 received Ambicor penile implants. All incontinent patients received a 4.0-cm cuff AMS 800 AUS. Dual implantation showed statistically significant reduction in the operative time when compared with the total time for the individual procedures. Furthermore, it was associated with approximately a $7000 cost savings compared with individual procedures. No prosthetic infections or erosions were encountered in this series.

Mancini et al. compared outcomes of postprostatectomy patients who underwent dual implantation (DI) to those receiving AUS or IPP alone from 2001 to 2006 using AMS 800 AUS and AMS 700CX IPP [10]. Telephone interviews using a standard questionnaire were conducted to evaluate prosthetic functionality, ease of use, and patient satisfaction. A total of 95 men were evaluated (31 for IPP alone, 31 for AUS alone, and 33 for DI) with a mean postoperative follow-up time of 32.0, 18.9, and 21.6 months, respectively. Daily pad usage decreased from 4.6 to 0.8 pads per day with AUS alone and 6.1 to 1.3 pads per day with DI. Patients were satisfied with IPP rigidity during inflation and flaccidity during inactivation in both IPP and DI groups in a similar manner. Overall prosthetic satisfactions as well as ease of scrotal pump operation were similar in all groups. The majority of patients stated that they would have the procedure done again (77% to 94%) or recommend the DI procedure to a friend or relative (87% to 94%). 

Infection has been always a concern for the operating surgeon since it may spread to all components necessitating their removal and making revision more challenging. Based on the above series, there appears to be a vast discrepancy between infection rates ranging from 0 to 11%. This in part can be explained by the number of cases reported per series, the experience of the operating surgeons, the difference in follow-up periods, and the advances in the prostheses being used. In particular, the largest series with no infections describes 105 patients implanted with antibiotic-impregnated prostheses [11], and this appears to have significantly lowered the infection rate. It is worth mentioning that if the 2 devices are compartmentalized then both devices do not have to be removed if one becomes infected.

Nevertheless, infected dual implants may still be amenable to salvage procedures as reported by Bryan et al. [23]. The authors reported their experience with removal, antiseptic irrigation, and immediate reimplantation of infected noneroded AUS in 8 patients. Three of the 8 patients underwent successful concurrent 3-piece IPP salvage as well. One patient with a dual implant underwent the dual salvage twice. All three salvage patients had originally received a simultaneous dual implant. Parulkar and Barrett stated that, in the event of infection, should one acts early to locate the components affected, it is possible to salvage the components of the unaffected device [7].

## 5. Conclusions

Concurrent urinary incontinence and ED are debilitating conditions and increasingly seen in a cohort of men especially the postradical prostatectomy population. AUS and the IPP are well-established treatments for these conditions when they are refractory to other less invasive measures. Dual insertion of an AUS and IPP, either synchronous or nonsynchronous, appears to be safe and efficacious offering long-standing solution to these problems. In addition, the availability of the single transverse scrotal incision has made synchronous insertion feasible with its attained cost and time benefits without additional morbidity. Synchronous dual implantation should be considered and offered to patients requiring both devices, thus avoiding the risk associated with a secondary procedure.

## Figures and Tables

**Table 1:** Comparison between advantage and disadvantage of synchronous and nonsynchronous dual prosthetic insertion [7–17].

Synchronous dual implantation	Sequential dual implantation
Advantages	Advantages

(i) Single incision (ii) Faster operating time (iii) Single anaesthetic event (iv) Shorter hospital stay (v) Decreased overall recovery time (vi) Increased cost savings (vii) Theoretical reduction in the infection risk (viii) Supine position allowing more mobility of the bulbar urethra facilitating posterior dissection (ix) Easier placement of the AUS pump (x) Decrease in the risk of AUS pump migration	(i) More time allowed for a patient to accommodate the first device before considering a second implant (ii) Theoretical decreased initial confusion to operate the device (iii) For AUS, there appears to be a higher completely dry rate and fewer subsequent tandem cuff additions when placed at a more robust proximal bulb of the urethra during a perineal incision

Disadvantages	Disadvantages

(i) Sufficient patient dexterity needed to activate either pump as required (ii) Theoretical concerns of patient confusion and difficulty of use with two scrotal pumping devices (iii) Surgeon experience and learning curve. Requires more experienced prosthetic urologists (iv) If the bulbar urethra is considered difficult through the scrotal incision, the surgeon should use the perineal incision for the cuff placement (v) Infection would potentially necessitate removal of both implants components (vi) Concerns over the extent of dissection which may further increase the risks of erosion and infection (vii) Distal placement of the AUS cuff on the thin urethra may be less effective, with a higher early failure rate and revisions due to loosely fitting cuffs and accelerated urethral atrophy	(i) Two incisions (ii) Previous prosthetic implant may have asymptomatic colonization of the prosthetic device predisposing to infection during insertion of the second implant (iii) Extra care is needed to avoid damaging the components of the existing implant and its tubing (iv) Added danger of operating in an area with surgical scarring (v) Insertion of the sphincter pump from an abdominal incision can be associated with excessive edema or hematoma causing the pump to retract into the upper groin, making long-term use by the patient difficult (high riding pump)
